# NSUN6-mediated 5-methylcytosine modification of NDRG1 mRNA promotes radioresistance in cervical cancer

**DOI:** 10.1186/s12943-024-02055-2

**Published:** 2024-07-05

**Authors:** Min Yu, Mengdong Ni, Fei Xu, Chaohua Liu, Lihua Chen, Jiana Li, Siyu Xia, Yixin Diao, Jiaxin Chen, Jun Zhu, Xiaohua Wu, Min Tang, Jiajia Li, Guihao Ke

**Affiliations:** 1 Department of Gynecologic Oncology, Fudan University Shanghai Cancer Center, Shanghai, 200032 China; 2 Department of Oncology, Shanghai Medical College, Fudan University, Shanghai, 200032 China; 3 Shanghai University of Traditional Chinese Medicine, Shanghai, 201203 China

**Keywords:** Cervical cancer, Radioresistance, m^5^C modification, NSUN6, 3D bioprinted patient-derived organoid

## Abstract

**Background:**

Radioresistance is the leading cause of death in advanced cervical cancer (CC). Dysregulation of RNA modification has recently emerged as a regulatory mechanism in radiation and drug resistance. We aimed to explore the biological function and clinical significance of 5-methylcytosine (m^5^C) in cervical cancer radiosensitivity.

**Methods:**

The abundance of RNA modification in radiotherapy-resistant and sensitive CC specimens was quantified by liquid chromatography-tandem mass spectrometry. The essential RNA modification-related genes involved in CC radiosensitivity were screened via RNA sequencing. The effect of NSUN6 on radiosensitivity was verified in CC cell lines, cell-derived xenograft (CDX), and 3D bioprinted patient-derived organoid (PDO). The mechanisms of *NSUN6* in regulating CC radiosensitivity were investigated by integrative m^5^C sequencing, mRNA sequencing, and RNA immunoprecipitation.

**Results:**

We found a higher abundance of m^5^C modification in resistant CC samples, and *NSUN6* was the essential m^5^C-regulating gene concerning radiosensitivity. NSUN6 overexpression was clinically correlated with radioresistance and poor prognosis in cervical cancer. Functionally, higher NSUN6 expression was associated with radioresistance in the 3D PDO model of cervical cancer. Moreover, silencing NSUN6 increased CC radiosensitivity in vivo and in vitro. Mechanistically, *NDRG1* was one of the downstream target genes of NSUN6 identified by integrated m^5^C-seq, mRNA-seq, and functional validation. NSUN6 promoted the m^5^C modification of *NDRG1* mRNA, and the m^5^C reader ALYREF bound explicitly to the m^5^C-labeled *NDRG1* mRNA and enhanced *NDRG1* mRNA stability. NDRG1 overexpression promoted homologous recombination-mediated DNA repair, which in turn led to radioresistance in cervical cancer.

**Conclusions:**

Aberrant m^5^C hypermethylation and NSUN6 overexpression drive resistance to radiotherapy in cervical cancer. Elevated NSUN6 expression promotes radioresistance in cervical cancer by activating the NSUN6/ALYREF-m^5^C-NDRG1 pathway. The low expression of NSUN6 in cervical cancer indicates sensitivity to radiotherapy and a better prognosis.

**Supplementary Information:**

The online version contains supplementary material available at 10.1186/s12943-024-02055-2.

## Background

Cervical cancer (CC) is one of the most common gynecological malignancies. Cervical cancer incidence and mortality have declined in developed countries following the advances in prevention and screening. However, these incidences and mortality rates remain high or have even increased in developing countries [1]. Radiotherapy is strongly recommended for advanced cervical cancer. However, radioresistance is the leading cause of cervical cancer-related deaths [2]. Approximately 30% of patients with advanced cervical cancer exhibited resistance to radiotherapy [3]. Therefore, it is urgent to unveil the underlying molecular mechanism and explore potential strategies to overcome radioresistance in cervical cancer. To date, several mechanisms of radioresistance have been studied, such as DNA damage response, hypoxia, tumor angiogenesis, tumor heterogeneity, cancer stem cells, immune invasion, inflammation, and metabolism [4–6]. Recent work also indicated epigenetic dysregulation may contribute to cancer cell radiation resistance [7–10].

RNA epi-modifications such as m^6^A decoration play critical roles in gene expression and dynamically regulate various physiological and pathological processes, such as tumorigenesis [11]. Another important RNA modification, 5-methylcytosine (m^5^C), was previously thought to be stable and highly abundant in non-coding RNAs such as tRNA and rRNA [12]. Based on advanced high-throughput techniques, recent studies have shown that m^5^C modification is also present in mRNA [13–16]. Recent discoveries verified that m^5^C is distributed in the 3’untranslated region (UTR), 5’UTR, and coding sequence (CDS) on mRNA [13]. NOP2/Sun RNA methyltransferase family members 2 and 6 (NSUN2 and NSUN6) have been characterized as writers that catalyze m^5^C deposition on mRNA [15, 16]. Aly/REF export factor (ALYREF) [16] and Y-box binding protein 1 (YBX1) [13, 17]have been identified as readers recognizing m^5^C-modified mRNA. Dynamic and reversible m^5^C modification regulates RNA metabolic processes, including RNA stability [13], export [16, 18], and translation [14].

The significance of m^5^C modification in malignancies has become an increasing focus of investigation. Evidence has shown that m^5^C hypermethylation is involved in tumorigenesis and drug resistance in bladder cancer [13], gastric cancer [19], esophageal cancer [20], non-small-cell lung cancer [17], and cervical cancer [21]. In addition, dysregulated expression of m^5^C-related enzymes such as NSUN6, NSUN2, and ALYREF has been reported in several cancers. NSUN6 [22], one of the m^5^C methyltransferases, plays a critical role in cell proliferation and tumor progression. Nevertheless, whether and how m^5^C modifications and NSUN6 play roles in cervical cancer radiosensitivity remain unclear.

By liquid chromatography-tandem mass spectrometry (LC-MS/MS) and mRNA-sequencing (mRNA-seq) analysis, we found that RNA m^5^C abundance and *NSUN6* expression were predominantly up-regulated in radioresistant CC samples. 3D bioprinted patient-derived organoid (PDO) and cell-derived xenograft model (CDX) further verified that elevated NSUN6 promotes resistance to radiotherapy in cervical cancer. Integration of methylated RNA immunoprecipitation sequencing (MeRIP-seq) and mRNA-seq analysis revealed that NSUN6 catalyzes N-myc downstream regulated 1(*NDRG1*) mRNA translation in a m^5^C-dependent manner. Genomic silencing of the NSUN6-m^5^C-NDRG1 pathway overcomes radioresistance in cervical cancer. Furthermore, we demonstrated that ALYREF stabilizes *NDRG1* mRNA by recognizing the m^5^C-modified site on its 3’UTR by RNA immunoprecipitation (RIP) sequencing analysis. Thus, our findings illustrate a regulatory mechanism of RNA m^5^C-mediated radioresistance and provide a therapeutic rationale for targeting the NSUN6/ALYREF-m^5^C-NDRG1 signaling axis in cervical cancer.

## Materials and methods

### The human specimens

Cervical cancer tissues were obtained from patients with advanced cervical cancer who received radical radiotherapy at Fudan University Shanghai Cancer Center (FUSCC) from January 2018 to December 2019. The pre-treatment tumor samples used in this study were obtained with informed consent from the patients and approved by the FUSCC Ethics Committee. Cervical cancer patients who do not achieve complete clinical response (CR) after initial radical radiotherapy or relapse within the radiation field within six months after CR are defined as radioresistant cases [23]. Patients whose recurrence interval exceeds six months are radiosensitive cases. In our study, all patients received platinum-containing concurrent radiotherapy with a follow-up time over six months. Primary radioresistant and radiosensitive cervical cancer cases were screened by retrospectively analyzing patient outcomes. Propensity matching analysis was used to control the effects of confounding factors such as age, tumor size, parametrial extension, lymph node metastasis, and stage in the radioresistant and radiosensitive group. Finally, 21 cases were included in each group for the follow-up study. The clinical information of these 42 patients was summarized in Supplementary Tables 1 and Supplementary Table 5.

### RNA modification quantification by LC-MS/MS

RNA modification quantification by LC-MS/MS was conducted as described previously [24]. Total RNAs were isolated from cervical cancer tissues using TRIzol reagent (Life Technologies). 200 ng RNA was incubated with nuclease P1 (Sigma-Aldrich) in 20 µl buffer containing 25 mM NaCl, 2.5 mM ZnCl_2_ at 37 °C for 2 h, then 2.2 µl NH_4_HCO_3_ (1 M), and alkaline phosphatase (Sigma-Aldrich) added, and the solution was then incubated again at 37 °C for 2 h. Following centrifugation at 13,000 rpm for 10 min at 4 °C, 10 µl of the solution was analyzed by LC-MS/MS at the Mass Spectrometry Application Research Center of the Institutes of Biomedical Sciences at Fudan University.

### Cell culture

SiHa, Me-180, MS751, and HEK293T cell lines used in this study were obtained from ATCC. SiHa, MS751, and 293T (Me-180) cells were cultured in DMEM (MyCo5A) medium with 10% FBS and 1% Penicillin-Streptomycin. STR authentication of these cell lines was carried out in November 2022. Real-time PCR was performed periodically to ensure that cells were free of mycoplasma contamination.

### Establishment of the 3D bioprinted PDO model

Biopsy samples were thoroughly rinsed with DPBS (Gibco) and cut into 3–5 mm pieces. The cut samples were transferred into digestion tubes filled with type I collagenase (YEASEN, Shanghai, China) and dissociated with a tissue dissociator (Cyberiad Biotech, Shanghai, China) for 10–15 min. The cell suspension was collected by centrifugation at 200 g for 5 min.

Cell suspensions containing 50 million cells per ml were prepared from dissociated tumor cells. Light-cured bioinks composed of 8.5% gelatin methacrylate (Yuju Technology, Shanghai, China), 0.5% hyaluronic acid methacrylate (Yuju Technology), and 0.2% lithium phenyl-2,4,6-trimethylbenzoylphosphonate (TCI Chemicals) were formulated for bioprinting. Bioprinting of patient-derived tissues (PDTs) was bioprinted using Biocube (Cyberiad Biotech, Shanghai, China), a high-throughput light-based bioprinter at a wavelength of 405 nm. The cell suspension was mixed with bioink in a 1:1 ratio before printing, and the mixture was added to a 96-well plate at 2µL/well. Then, the mixture was cured using a bioprinter for 25 s with a light intensity of 50%. Afterward, the bioprinted constructs were rinsed with DPBS and cultured in a maintenance medium at 37 °C and 5% CO2. CellTiter-Glo 3D Cell Viability Assay (Promega, G9683) was used to examine the cell viability in 3D organoids.

### In vitro cell proliferation, colony formation, cycle arrest, and apoptosis assays

For the cell proliferation assay, 1000 cells were seeded into 96-well plates, and cell viability was assessed using the CCK-8 kit (YEASEN). For the colony formation assay, 1000 cells were seeded into 6-well plates and cultured continuously for 14 days, stained with crystal violet, and then counted for the number of clones. Cell cycles and apoptosis were analyzed using flow cytometry. For the cell cycle analysis, cells were stained with propidium iodide/RNase (BD Pharmagen, USA). For the apoptosis assay, cells were stained using PE Annexin V/7-AAD reagent (YEASEN).

### siRNA, plasmid, and lentivirus

siRNAs targeting NSUN6 and NDRG1 (Supplementary Table 3) were purchased from Genepharma (Shanghai, China). Cells were transfected using the Hieff Trans siRNA/miRNA transfection reagent (YEASEN).

shRNAs targeting NSUN6 (0081-221078), ALYREF (310-10189), and the plasmid overexpressing NSUN6 (310-221078) and ALYREF (0081-10189) were purchased from lncbio (Shanghai, China), NDRG1 overexpressing plasmid (54734GV) was purchased from Genomeditech (Shanghai, China). HEK293T cells were used for lentivirus production. For HEK293T cells in a 10-cm dish, 8 µg of plasmid DNA, 6 µg of psPAX2 (Addgene), 2.4 µg of pMD2.G (Addgene), and 24 µl of Polyethylenimine Linear (YEASEN) were mixed. The supernatant liquid was collected 48 h post-transfection to infect cervical cancer cells.

### RNA extraction, quantitative real‑time PCR (qRT‑PCR)

Total RNA was extracted using TRIzol reagent (Invitrogen, Carlsbad, USA). Total RNA (1 µg) was reverse-transcribed to cDNA using a PrimeScript RT kit (Takara Bio Inc., Japan). qRT-PCR was performed using a SYBR Green kit (Takara Bio Inc., Japan). The Actin, NSUN6, NDRG1, and ALYREF primers are listed in Supplementary Table 4.

### Analysis of NDRG1 mRNA stability

Cells were collected at 0, 3, 6, and 9 h after treating actinomycin D (Act D, 4 µg/ml). RNA was isolated from these cells for qRT-PCR.

### HR and NHEJ reporter assay

Firstly, HeLa cells stably expressing homologous recombination (HR) and non-homologous end joining (NHEJ) reporter systems were constructed. Then, Hela HR and NHEJ reporter cells in the 6-cm dish were transfected with NDRG1 siNC and siRNA, respectively (CTRL and oeNDRG1 plasmid). 24 h later, 2 µg of I-SceI plasmid was transfected into the cells. 48 h later, cells were collected and the proportion of GFP-positive cells was analyzed by flow cytometry.

### Purified chromatin samples (chr)

Cell precipitates were resuspended in 200 µl of Buffer 1 (6 cm dish) (150 mM NaCl, 50 mM Hepes 7.5, 1 mM EDTA), 0.1% Triton X-100, Protease Inhibitor cocktail (Roche) for 3 min on ice. Lysates were precipitated at 13,000 rpm for 3 min. The insoluble precipitate was washed twice in buffer 1 without Triton X-100, resuspended in 100 µl of buffer 2 (150 mM NaCl, 50 mM Hepes 7.5, 1 mM EDTA, 200 µg/ml RNaseA, protease inhibitor cocktail (Roche)), and incubated for 30 min at 25 °C. The samples were centrifuged for 3 min at 13, 000 rpm, and the remaining precipitate (purified chromatin samples (Chr)) was resuspended in SDS loading buffer, boiled, and sonicated for solubilization prior to WB analysis.

### Western blotting analysis (WB)

Proteins were separated with SDS-PAGE and transferred to the PVDF membrane. The following antibodies were analyzed: NSUN6 (1:1000, 17240-1-AP, Proteintech), γH2AX (1:1000, 9718, CST), NDRG1(1:1000, 26902-1-AP, Proteintech), ALYREF (1:1000, ab6141, Abcam), RAD51 (1:1000, 67024-1-Ig, Proteintech), ATR (1:1000, 2790, CST), pATR(ser428) (1:1000, 2852, CST), pBRCA1(ser1524) (1:1000, 9009, CST), CHK1 (1:1000, 2360, CST), pCHK1(ser345) (1:1000, 2341, CST), Histone H3 (1:1000, 4499, CST), GAPDH (1:10000, 60004-1-Ig, Proteintech), and β-tubulin (1:10000, 10068-1-AP, Proteintech). HRP-conjugated anti-rabbit or anti-mouse (CST) were used as secondary antibodies.

### Immunofluorescence (IF)

Cells (or organoids) were seeded on coverslips in 24-well plates, fixed with 4% paraformaldehyde overnight at 4 ℃, and then permeabilized with 0.5% Triton X-100 in PBS for 10 min (organoids for 30 min). After blocking in 5% BSA for 1 h, the cells or organoids were sequentially incubated with primary and corresponding secondary antibodies. Finally, the nuclei were stained with DAPI. Antibodies used in the IF included NSUN6 (1:100, 17240-1-AP, Proteintech), γH2AX (1:200, ARG55251, Arigobio), NDRG1(1:100, 26902-1-AP, Proteintech), and RAD51 (1:100, 67024-1-Ig, Proteintech).

### Multiplex immunofluorescence (multi-IF)

Paraffin-embedded tissue sections of CC were deparaffinized in xylene and then rehydrated sequentially in 100%, 95%, 70% ethanol, and PBS buffer. For IF, sections were blocked with 5% goat serum in PBS buffer, then incubated overnight with NSUN6 antibody (1:100, 17240-1-AP, Proteintech) at 4℃ and incubated with corresponding HRP secondary antibody for 1 h at room temperature. Sections were incubated with tyramide signal amplification (TSA) (Servicebio Technology Co., Ltd. Wuhan, China) for 10 min at room temperature, then antigen repair and sealing were performed again. The Ki67 antibody (1:100, 27309-1-AP, Proteintech), corresponding HRP-labeled antibody, and TSA were sequentially added and incubated. Finally, the nuclei were stained with DAPI, and the images were scanned after the slices were sealed.

### Immunohistochemical staining (IHC)

IHC staining results were assessed independently by two pathologists. The intensity of staining was categorized as negative (0), weak (1), moderate (2), and strong (3). The final IHC staining score equals the product of the staining intensity and the percentage of positively stained tumor cells. The following antibodies were used for IHC: NSUN6 (1:1000, 17240-1-AP, Proteintech), NDRG1(1:1000, 26902-1-AP, Proteintech), Ki67 (1:1000, 27309-1-AP, Proteintech), γH2AX (1:200, 9718, CST) and RAD51 (1:500, 14961-1-AP, Proteintech).

### Establishment of CDX model

A subcutaneous transplanted model was used to evaluate the growth of SiHa shNC, SiHa shNSUN6, and SiHa shNSUN6-oeNDRG1 cells with or without radiation. Cells (7.5*10^6 per mouse, *n* = 6 for each group) were suspended in 100 µl PBS and subcutaneously inoculated into 5-week-old female Balb/C-nude mice (Gempharmatech Co.Ltd, China). After the tumors formed, the tumor volumes were measured once a day. Tumor volume was calculated using the formula: Volume = 0.5 × Length × Width 2 (mm^3^). When the volume reached 200 mm^3^, mice in the irradiated group were exposed to X-rays (doses: 16 Gy/2Fx). Mice were sacrificed when the tumor diameter reached roughly 1.5 cm, and the tumors were excised and weighed. Ethical approvals for the animal experiments were obtained from the FUSCC Ethics Committee.

### RNA sequencing, m^5^C sequencing and m^5^C-MeRIP‑qPCR

Total RNA was extracted from 20 radioresistant CC tissues and 19 radiosensitive CC tissues using the TRIzol reagent (Invitrogen, Carlsbad, USA). Transcriptome sequencing was performed by LC-Bio Technology CO., Ltd (Hangzhou, China).

Intact mRNA was first isolated from total RNA samples using an mRNA isolation kit per the manufacturer’s protocol (Promega). MeRIP was performed as previously described [24]. The isolated mRNA was chemically fragmented into 200 nucleotide-long fragments by incubating at 94 °C for 5 min. Then, m^5^C methylated mRNA was immunoprecipitated with an anti-m^5^C antibody. The bound RNAs were washed and eluted through competition with m^5^C, then purified by the RNA cleanup kit. Afterward, eluted RNA and MeRIPed RNA were analyzed by deep sequencing on an Illumina Hiseq instrument at the Cloud-Seq Biotech Inc. (Shanghai, China). MeRIP-qPCR also analyzed the immunoprecipitated samples. The specific primers are provided in Supplementary Table 4.

### RNA immunoprecipitation (RIP)-PCR

RIP assays were performed using the MagnaRIP RNA-binding protein immunoprecipitation kit (Millipore). Briefly, cell lysates were incubated with magnetic beads conjugated with 5 µg of anti-ALYREF antibody by spinning at 4 °C overnight. Then, the RNA-protein-beads complexes were washed and eluted with proteinase K buffer. RNA in the mixture was extracted using the phenol-chloroform RNA extraction method. Finally, the relative expression of ALYREF-enriched RNA was determined by qPCR and normalized to the input.

### Statistical analysis

GraphPad Prism software (version 10.1.1) were used for data analysis, and student’s t-test or ANOVA was used for group comparisons. overall survival (OS) and recurrence-free survival (RFS) were compared using the log-rank test and were plotted by the SRplot online platform (bioinformatics.com.cn/). NSUN6 and NDRG1 expression levels were dichotomized according to the optimal cutoff value calculated by the KMplot database (kmplot.com) before performing the log-rank test. A *p*-value < 0.05 (two-tailed) was considered statistically significant.

## Results

### m^5^C hypermethylation and NSUN6 overexpression are correlated with radioresistance in cervical cancer

**Fig. 1:**
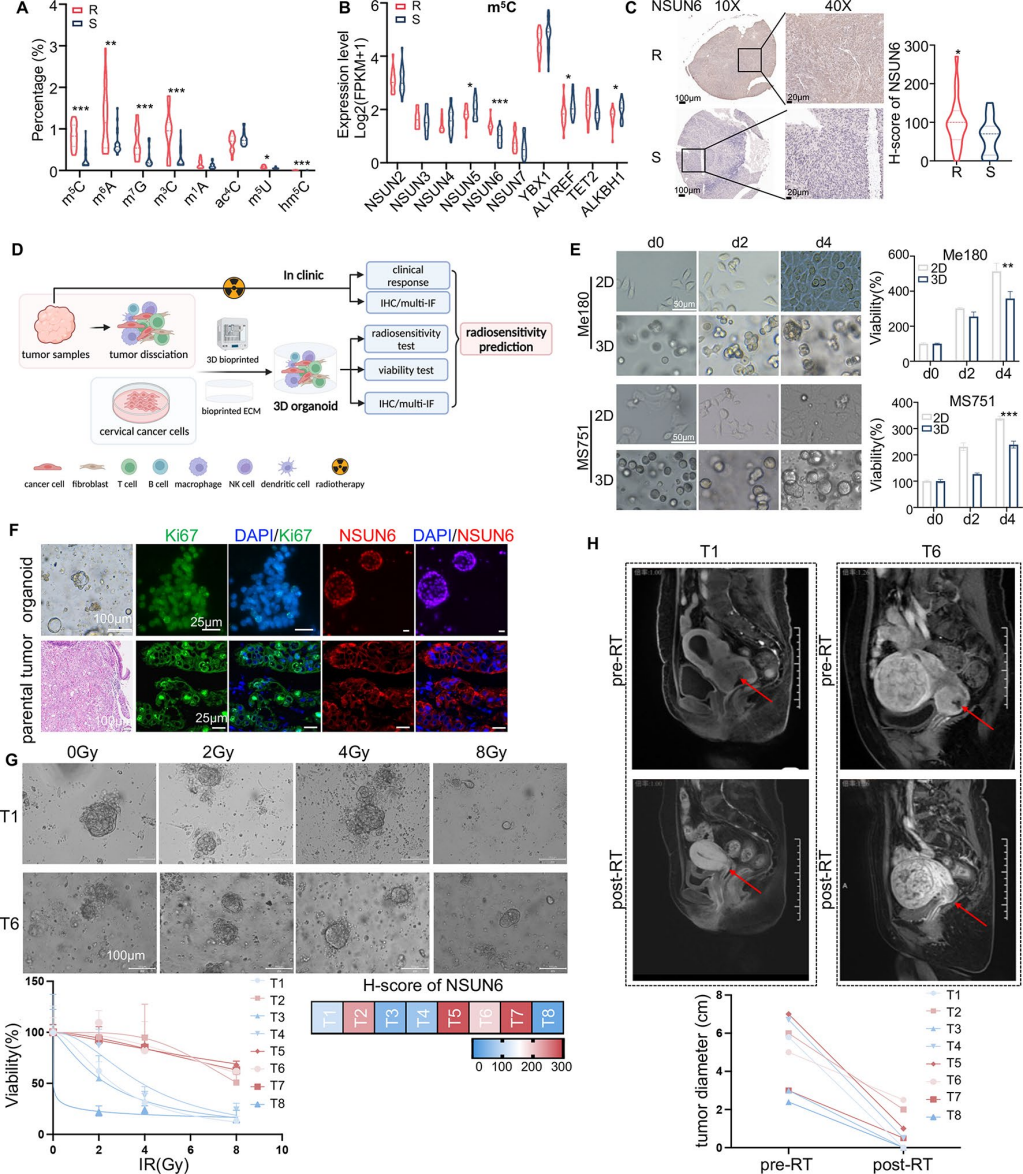
RNA m^5^C hypermethylation and NSUN6 overexpression correlate with radioresistance in cervical cancer. (**A**) Quantifying RNA modifications by mass spectrometry in radioresistant and radiosensitive CC samples. R, radioresistant samples; S, radiosensitive samples; m^5^C, 5-methylcytosine; m^6^A, *N*^6^-methyladenosine; m^7^G, 7-methylguanosine; m^3^C, 3-methylcytidine; m^1^A, *N*^1^-methyladenosine; ac^4^C, *N*^4^-acetylcysteine; m^5^U, 5-methyluridine; hm^5^C, 5-hydroxymethylcytosine. (**B**) The expression of 10 m^5^C regulators between radioresistant and radiosensitive CC samples was detected by mRNA sequencing. (**C**) Representative immunohistochemical staining (IHC) images of NSUN6 protein levels in radioresistant and radiosensitive samples. Scale bar, 100 μm (left) and 20 μm (right). (**D**) Flowchart of 3D-bioprinting cervical cancer organoids predicting radiosensitivity for cervical cancer. The diagram was created using BioRender. (**E**) Monolayer growth images of Me-180 and MS751 cells in 2D and spherical growth images in 3D models at days 0, 2, and 4, respectively (left). Scale bar, 50 μm. Cell viability of Me-180 and MS751 in 2D and 3D models on days 0, 2, and 4 (right). (**F**) Immunofluorescence staining (IF) images for NSUN6 and Ki-67 of cervical cancer samples and corresponding 3D organoids. (**G**) Dose-response to radiation of 3D bioprinted CC organoids. Representative bright-field images on day four after irradiation in two selected cases (top). Dose-response curves on day four after irradiation in eight cases (left). NSUN6 expression in these eight samples by IHC staining (right). (H) MRI images of the tumors before and one month after radiotherapy (top). Tumor diameter changes before and after radiotherapy for these eight patients (bottom). A two-sided Student’s t-test was used for statistical analysis (A-C, E, and G). *p < .05, **p < .01, ***p < .001, error bars represent means ± SD

To elucidate the functional roles of epi-transcriptome in cervical cancer radiosensitivity, we first examined the abundance of RNA modifications in 21 radioresistant CC tissues and 21 paired radiosensitive CC tissues by LC-MS/MS assay, including 5-methylcytosine (m^5^C), *N*^6^-methyladenosine (m^6^A), 7-methylguanosine (m^7^G), 3-methylcytidine (m^3^C), *N*^1^-methyladenosine (m^1^A), *N*^4^-acetylcysteine (ac^4^C), 5-methyluridine (m^5^U) and 5-hydroxymethylcytosine (hm^5^C). Interestingly, we found that the modification levels of RNA m^5^C, m^6^A, m^7^G, and m^3^C were significantly up-regulated in resistant samples but down-regulated in sensitive samples (Fig. 1A). RNA modifications are dynamically regulated by RNA-modifying proteins (RMPs, writers, erasers, and readers). Therefore, we speculated that the dysregulation of RMPs causes the elevated level of RNA methylation in radioresistant cervical cancer. To investigate the critical RNA-modifying genes regulating radiosensitivity in cervical cancer, we performed transcriptome sequencing on 20 resistant and 19 sensitive samples (Fig. S1A, B). By analyzing the mRNA-seq data, we observed down-regulation of two readers (*ALYREF* and *YTHDF1*), one writer (*NSUN5*), and one eraser (*ALKBH1*) in radioresistant samples compared with sensitive samples (Fig. 1B and Fig. S1C, D). The mRNA level of m^5^C methyltransferase *NSUN6* significantly increased in radioresistant CC tissues compared with that in radiosensitive tissues (Fig. 1B). Consistently, the protein expression levels of NSUN6 were found to be noticeably higher in radioresistant CC samples than in the radiosensitive samples by IHC staining (Fig. 1C). The above results implied that m^5^C level and NSUN6 expression were consistently upregulated in the radioresistant CC samples.

#### NSUN6 overexpression indicates radioresistance in patients-derived 3D bioprinted CC organoids

Recent studies have demonstrated the potential of organoids to predict clinical drug responses [25, 26]. However, the capacity of these organoids to forecast radiosensitivity in cervical cancer remains an unresolved issue. We constructed a 3D bioprinted PDO model to validate the clinical response of cervical cancer to irradiation (IR) (Fig. 1D).

we first constructed the Me-180 or MS751 cells-derived 3D bioprinted organoids and then tested the consistency of cell proliferation and radiation response in both 2D and 3D models (Fig. 1E and Fig. S2A). Consistent with previous studies [27], we observed spherical growth of Me-180 and MS751 cells in 3D models and monolayer growth in 2D models on day four after bioprinting (Fig. 1E). As shown in Fig. 1E and Fig. S2A, we found that the proliferation capacity and radiation response were highly consistent in both 2D and 3D models. However, we also observed that the spheroids in a 3D culture were more sensitive to IR than cells in a conventional 2D culture (Fig. S2A), which may be caused by the inhibitory effect of the bioinks used for 3D bioprinting on cell proliferation.

The results encouraged us to construct a 3D bioprinted CC-PDO model. Cervical cancer biopsy samples were obtained from patients with newly diagnosed advanced cervical cancer. Tumor cells were isolated and cultured with the light-cured bioinks described in the previous study. Eight CC organoids were established and expanded for multi-IF staining and radiosensitivity testing. By analyzing the multi-IF images, we found that organoids and paired primary tumor tissues exhibited similar staining patterns for NSUN6 and Ki-67 (Fig. 1F). Overall, the data suggested that 3D organoids recapitulate molecular features of parental tissues with high consistency. To validate the response of CC organoids to IR in vitro, a quantitative comparison of 3D-cell viability and size changes in eight PDOs treated with 0, 2, 4, and 8 Gy IR was performed (Fig. 1G). The dose-response curves demonstrated individual differences in radiosensitivity among different PDOs. For example, organoids derived from T1 and T6 were sensitive and resistant to IR, respectively (Fig. 1G). To further verify the relationship between the expression of NSUN6 and the radiosensitivity of the organoids, the NSUN6 protein level in primary tumor samples was analyzed by IHC staining (Fig. S2B). Based on the IHC staining, the eight organoids could be categorized into two groups with high or low NSUN6 expression (Fig. 1G and Fig. S2B). As expected, cervical cancer organoids with low NSUN6 expression were more sensitive to IR than those with high NSUN6 expression(Fig. 1G). To assess the ability of 3D PDOs to predict radiation responses in cervical cancer patients, we also evaluated the clinical radiotherapy responses of these eight cervical cancer patients by MRI examination (Fig. 1H). Patients (T1, 3, 4, and 8) whose PDOs were sensitive to IR had higher rates of clinical complete responses than patients (T2, 5, 6, and 7) whose PDOs were resistant to IR. These results indicated that NSUN6 overexpression correlates with radioresistance, and 3D PDOs can predict patients’ clinical responses to radiotherapy in cervical cancer.

### NSUN6 knockdown sensitizes cervical cancer to radiotherapy

**Fig. 2:**
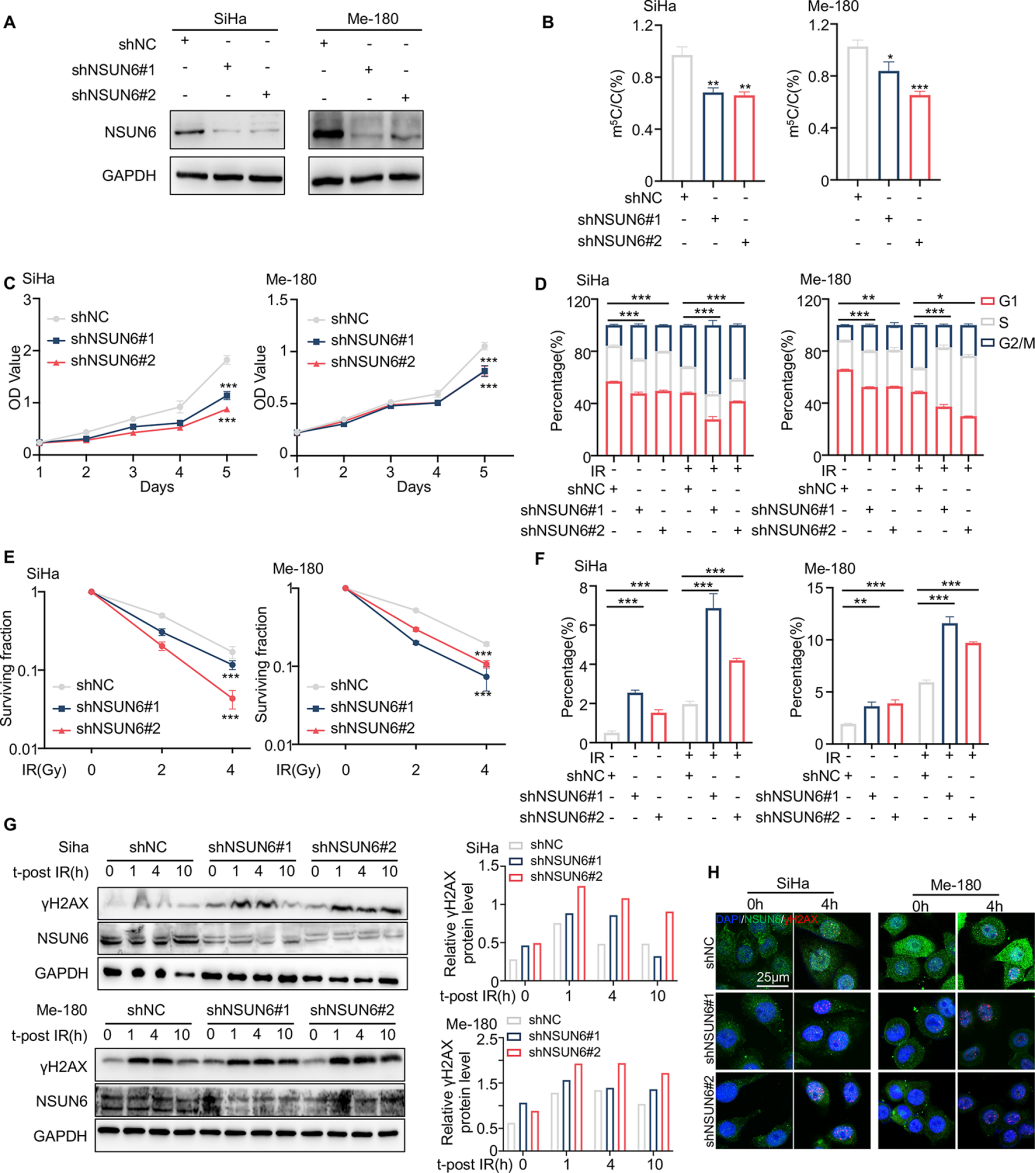
Downregulation of NSUN6 enhances the radiosensitivity of cervical cancer. (**A**) Western blotting detected NSUN6 levels in SiHa and Me-180 knockdown cells. (**B**) LC-MS/MS measured the RNA m^5^C levels in SiHa and Me-180 cells with NSUN6 knockdown. (**C**) Proliferation curves of NSUN6 knockdown cells and corresponding negative control (NC) cells. (**D**) The histogram of cell cycle distribution of NSUN6 knockdown cells and corresponding NC cells with or without IR. (**E**) Dose-response to irradiation of NSUN6 knockdown cells and corresponding NC cells. The surviving fraction was analyzed using a colony formation assay. (**F**) The percentage of apoptosis of NSUN6 knockdown cells and corresponding NC cells with or without IR. (**G**) Western blotting detected dynamic changes in γH2AX expression after irradiation (4 Gy) (left). Quantification of relative γH2AX protein levels using Image J software (right). Western blots do not show error bars because they represent a single measurement. (**H**) Representative images of γH2AX foci formations at 0 and 4 h after irradiation (4 Gy). Scale bar, 25 μm. A two-sided Student’s t-test was used for statistical analysis (B-F). *p* < .05, **p < .01, ***p < .001, error bars represent means ± SD. m^5^C, 5-methylcytidine; C, cytidine; IR, irradiation

To determine whether inhibiting NSUN6 could sensitize cervical cancer to radiotherapy, we established stable NSUN6-knockdown cervical cancer cells (SiHa and Me-180) for loss-of-function studies (Fig. 2A and Fig. S2A). As expected, the RNA m^5^C level was dramatically reduced on NSUN6 knockdown (Fig. 2B). We further evaluated the proliferation and radiosensitivity of NSUN6-knockdown cells. The CCK-8 assay showed that downregulation of NSUN6 suppressed the cells’ proliferation ability (Fig. 2C). NSUN6 knockdown induced cell cycle arrest after IR (Fig. 2D). Moreover, stable knockdown of NSUN6 reduced the clonogenic ability and increased apoptosis of SiHa and Me-180 cells following radiation exposure (Fig. 2E, F, and Fig. S2D). Results of clone formation assay and apoptosis assay indicated that NSUN6 knockdown decreased the IR dose threshold for cervical cancer cell death. Western blot analysis showed that the expression of γH2AX, a biomarker of DNA damage, was persistently present in NSUN6 knockdown cells after IR (Fig. 2G). Additionally, IF staining revealed the presence of more γH2AX positive foci in NSUN6-downregulated cells following IR (Fig. 2H). The findings suggested that reducing NSUN6 expression can enhance cervical cancer radiosensitivity in vitro. In addition, we found that downregulating NSUN6 increased the sensitivity of cervical cancer cells to cisplatin/Olaparib (Fig. S2E, F).

#### NSUN6-mediated m^5^C modification of NDRG1 mRNA maintains its ALYREF-mediated stability

**Fig. 3:**
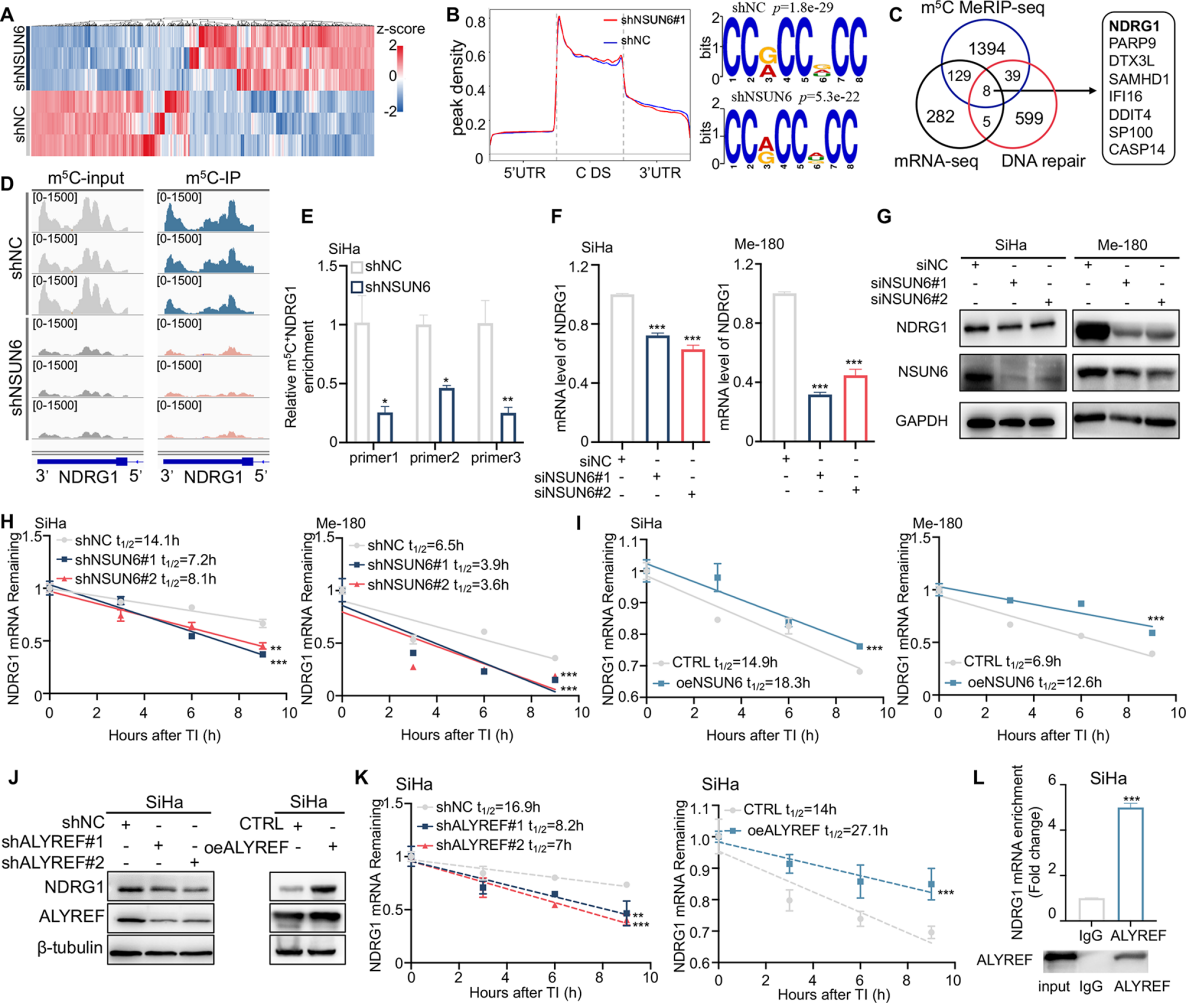
NSUN6 catalyzes m^5^C modification of NDRG1 mRNA. (**A**) The heatmap of differentially expressed genes in NSUN6 knockdown SiHa cells and corresponding NC cells. (**B**) The distribution of m^5^C modification on mRNA transcripts was identified by m^5^C-seq (left). The m^5^C consensus sequence motif in SiHa cell (right). (**C**) Integration of the mRNA-Seq, m^5^C-seq results, and DNA repair genes revealed that eight genes are consistently downregulated upon NSUN6 knockdown. (**D**) The IGV analysis showed changes in expression level and m^5^C abundance of NDRG1 mRNA upon NSUN6 knockdown. (**E**) MeRIP-qPCR analysis was employed to demonstrate NSUN6-mediated NDRG1 m^5^C modification in NSUN6 knockdown cells and corresponding NC cells. (**F**-**G**) The expression of NDRG1 upon NSUN6 knockdown was evaluated by RT-qPCR (F) and western blotting (**G**). (**H**-**I**) RT-qPCR detection of the half-life of NDRG1 mRNA in NSUN6 knockdown, NSUN6 overexpressing, and the corresponding control cells. Cells were treated with actinomycin D (4 µg/mL) for 3, 6, and 9 h. (**J**) The protein level of NDRG1 in ALYREF-knockdown and ALYREF-overexpressing SiHa cells was examined by western blotting. (**K**) The half-life of NDRG1 mRNA in ALYREF-knockdown and ALYREF-overexpressing SiHa cells. (**L**) The interaction between ALYREF protein and NDRG1 mRNA was detected by the RNA immunoprecipitation (RIP) assay using ALYREF-specific antibody and IgG control antibody. A two-sided Student’s t-test was used for statistical analysis (**F**, **H**-**I**, **K**-**L**). *p* < .05, **p < .01, ***p < .001, error bars represent means ± SD. CDS, coding sequence; UTR, untranslated regions; StopC, stop codon; StartC, start codon

**Fig. 4:**
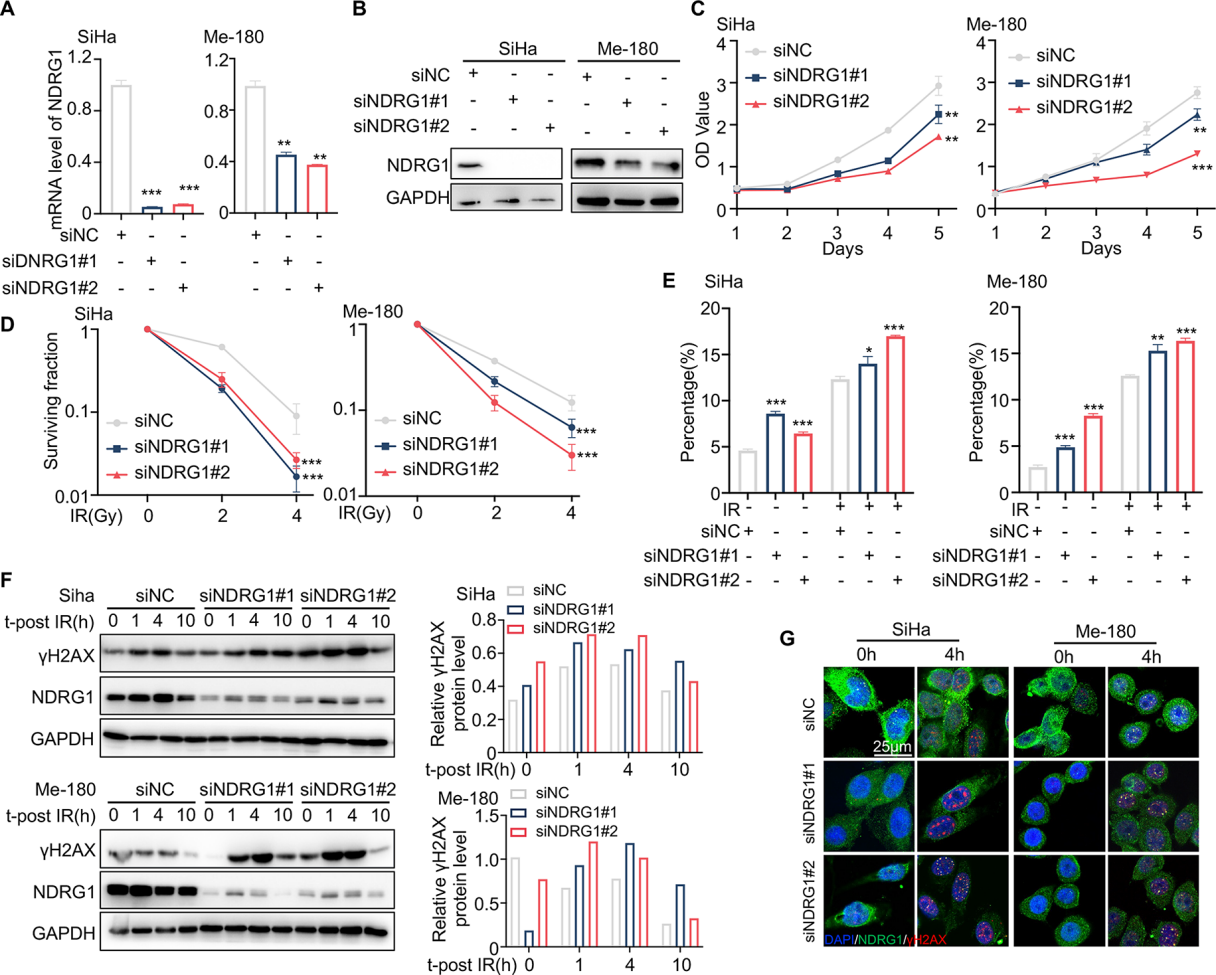
Silencing NDRG1 sensitizes cervical cancer to radiotherapy. (**A**-**B**) The efficiency of NDRG1 knockdown in SiHa and Me-180 cells was verified by RT-qPCR (A) and western blotting (**B**). (**C**) Proliferation curves of NDRG1 knockdown cells and corresponding NC cells. (**D**) Dose-response to irradiation of NDRG1 knockdown cells and corresponding NC cells. The surviving fraction was analyzed using the colony formation assay. (**E**) The percentage of apoptosis of NDRG1 knockdown cells and corresponding NC cells with or without IR. (**F**) Western blotting detected dynamic changes in γH2AX expression after irradiation (4 Gy) (left). Quantification of relative γH2AX protein levels using Image J software (right). Western blots do not show error bars because they represent a single measurement. (**G**) Representative images of γH2AX foci formations at 0 and 4 h after irradiation (4 Gy). Scale bar, 25 μm. A two-sided Student’s t-test was used for statistical analysis (**A**, **C**-**E**). *p < .05, **p < .01, ***p < .001, error bars represent means ± SD. IR, irradiation

The findings presented in Figs. 1 and 2B indicate that the pro-radioresistance of NSUN6 in cervical cancer may be dependent on its m^5^C catalytic activity. To elucidate the m^5^C-modified genes mediated by NSUN6 and to further elucidate the molecular mechanisms by which NSUN6 regulates radiosensitivity in cervical cancer, we performed mRNA and m^5^C-MeRIP sequencing in SiHa cells with or without NSUN6 knockdown. The mRNA-seq data revealed that 424 transcripts were significantly downregulated and 497 were upregulated after NSUN6 knockdown (Fig. 3A and Fig. S3A). Based on the gene ontology (GO) analysis conducted on Metascape (metascape.org), most downregulated genes showed enrichment in pro-oncogenic signaling pathways, including angiogenesis, MAPK, and hypoxia (Fig. S3B). MeRIP results showed that m^5^C sites on mRNA are distributed in the CDS (52%), StartC (19%), StopC (14%), 5’UTR (11%), and 3’UTR regions (4%) (Fig. S3C). Additionally, MeRIP-seq data showed that NSUN6-mediated m^5^C modification appears in the “CDCC (D = U, A or G)” consensus sequence and NSUN6 knockdown predominantly reduced m^5^C peaks in the 3’UTR region (Fig. 3B), consistent with the previous study. As expected, we found that the knockdown of NSUN6 significantly reduced the number of m^5^C peaks on the transcripts of SiHa cells (Fig. S3D). Enhanced DNA repair is a significant cause of radioresistance in cancer cells [28]. Integrated analysis of sequencing data and DNA repair-related genes [29] unveiled that eight genes with 13 peaks were simultaneously reduced upon NSUN6 knockdown (Fig. 3C and Supplementary Table 2). Among these eight downstream targets of NSUN6, NDRG1 (N-myc downstream-regulated gene 1) [30] was the top downregulated gene in response to shNSUN6 (Supplementary Table 2). Integrative Genomics Viewer (IGV) analysis showed that the mRNA expression and m^5^C level of NDRG1 significantly decreased in NSUN6-knockdown SiHa cells (Fig. 3D). MeRIP-qPCR further confirmed that NSUN6 knockdown decreased the m^5^C abundance of NDRG1 mRNA (Fig. 4E). Meanwhile, we found that both mRNA and protein levels of NDRG1 were downregulated in SiHa and Me-180 cells with NSUN6 knockdown (Fig. 3F, G). As the m^5^C site of NDRG1 mRNA is located in its 3’UTR (Fig. 3D), we speculated that NSUN6-mediated m^5^C modification may promote NDRG1 expression by enhancing NDRG1 mRNA stability. By RNA half-life assay, we discovered that knocking down NSUN6 significantly shortened the half-life of NDRG1 mRNA, while overexpressing NSUN6 prolonged the half-life of NDRG1 mRNA (Fig. 3H, I). Collectively, we clearly demonstrated that NDRG1 is a downstream target gene of NSUN6, and NSUN6 maintains NDRG1 mRNA stability in a m^5^C-dependent manner.

It is well known that RNA methylation regulates its target RNA by RNA recognition proteins [31]. Since m^5^C modification can stabilize NDRG1 mRNA, we first explored the effect of the known nuclear reader ALYREF on NDRG1 mRNA. Using the GEPIA database (gepia2.cancer-pku.cn), we found that ALYREF expression was significantly elevated in cervical cancer tissues and positively correlated with NSUN6 expression (Fig. S3E). In addition, we confirmed that silencing ALYREF inhibited NDRG1 mRNA and protein levels while overexpressing ALYREF elevated NDRG1 mRNA and protein levels (Fig. 3J and Fig. S3F). Moreover, the RNA half-life assay further demonstrated that silencing (overexpressing) ALYREF promoted (inhibited) NDRG1 mRNA degradation (Fig. 3K). Next, the specific interaction of ALYREF and DNRG1 mRNA was verified by RIP assay (Fig. 3L). Our findings indicated that NSUN6-mediated m^5^C modification maintains NDRG1 expression via ALYREF-dependent NDRG1 mRNA stability.

#### Silencing NDRG1 increases radiosensitivity in cervical cancer

NDRG1 is a crucial determinant of drug resistance by enhancing DNA repair [32, 33]. To investigate the effects of NDRG1 in cervical cancer radiosensitivity, we used small interference RNA (siRNA) treatment to silence NDRG1 expression in SiHa and Me-180 cells for functional test (Fig. 4A, B). Cell proliferation assay showed that transient NDRG1 knockdown significantly reduced the proliferation of human cervical cancer cells (Fig. 4C). Meanwhile, we observed that downregulation of NDRG1 attenuated the clonogenic ability and promoted apoptosis of SiHa and Me-180 cells following radiation exposure (Fig. 3D, E). Next, we assessed the role of NDRG1 in DDR using WB and IF assays to detect γH2AX expression after IR (Fig. 4F, G). WB results showed that γH2AX expression increased and persisted after irradiation in NDRG1-deficient cells (Fig. 2F). Additionally, IF staining revealed that downregulation of NDRG1 promotes the formation of γH2AX positive foci after irradiation (Fig. 2G). These results indicated that the ablation of NDRG1 sensitizes cervical cancer to radiotherapy by promoting IR-induced apoptosis and DNA damage.

#### NSUN6-NDRG1 axis is indispensable for DNA damage repair in an HR bias manner

**Fig. 5:**
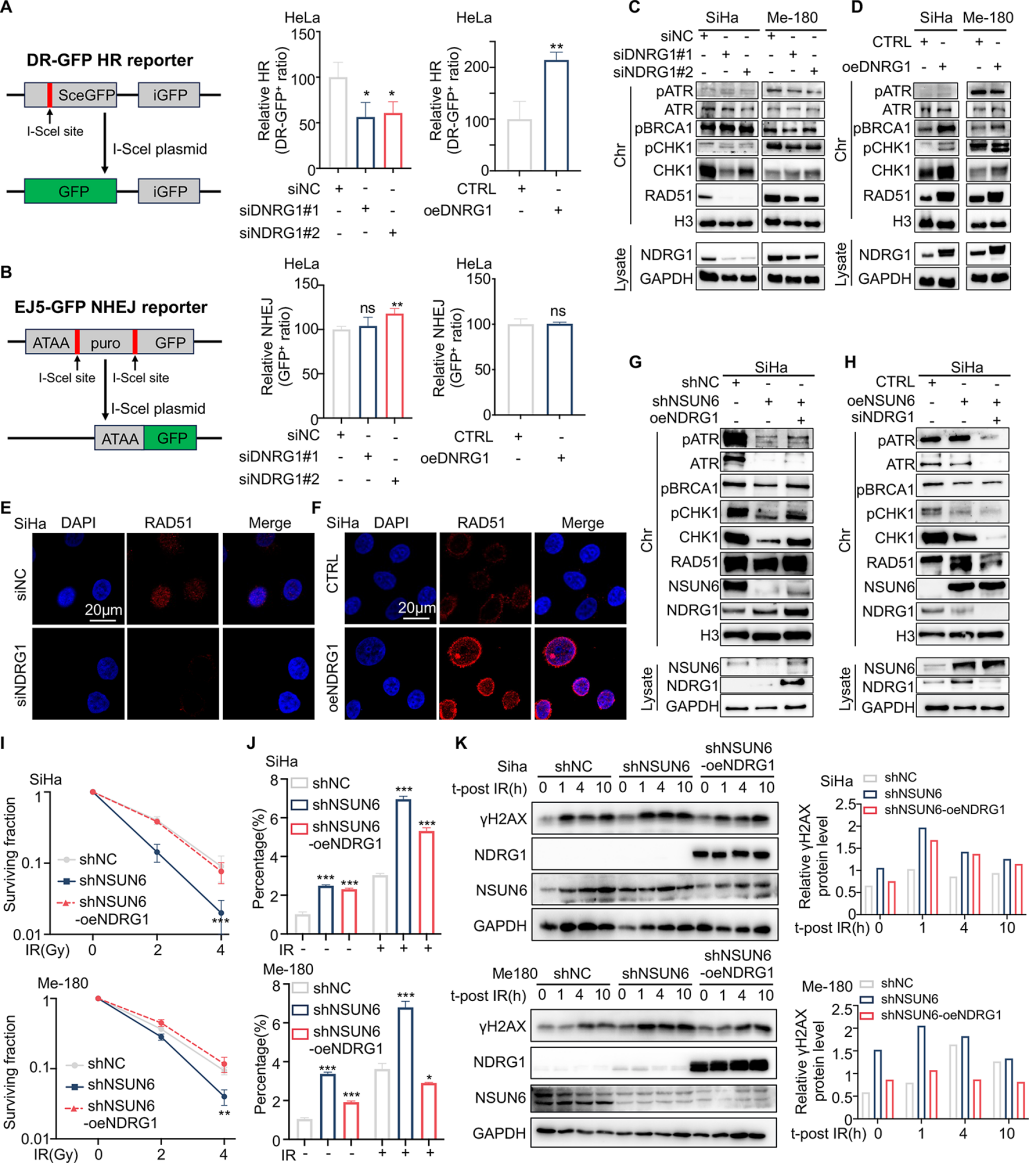
NDRG1 overexpression enhances HR-mediated DNA damage repair and rescues NSUN6 downregulation-induced radiosensitization in cervical cancer. (**A**-**B**) HR or NHEJ efficiency after knockdown or overexpression of NDRG1 in HeLa cells. (**C**-**D**) Western blotting detected the expression level of HR proteins bound to chromatin after knocking down or overexpressing NDRG1. (**E**-**F**) Representative images of RAD51 foci after silencing or overexpressing NDRG1. (**G**-**H**) Western blotting detected the expression level of HR proteins bound to chromatin in shNSUN6 cells, shNSUN6-oeNDRG1 cells, and control cells. (**I**-**J**) dose-response to irradiation (**I**), and apoptosis with or without irradiation (**J**). (**K**) Western blotting examined the dynamic expression of γH2AX after irradiation (4 Gy) in shNSUN6 cells, shNSUN6-oeNDRG1 cells, and control cells (left). Quantification of relative γH2AX protein levels using Image J software (right). Western blots do not show error bars because they represent a single measurement. A two-sided Student’s t-test was used for statistical analysis (A-B, I-J). *p < .05, **p < .01, ***p < .001, error bars represent means ± SD. IR, irradiation

Recent research suggests that NDRG1 stabilizes DDR proteins by direct protein-protein interaction [32, 33]. To explore whether NDRG1 is engaged in the DDR pathway and thereby regulates cervical cancer radiosensitivity, we examined the role of NDRG1 in homologous recombination (HR) and non-homologous end joining (NHEJ) by performing I-Secl-based HR and NHEJ reporter assays, respectively. As shown in Fig. 5A and B, NDRG1 downregulation (upregulation) significantly inhibited (increased) the efficiency of HR rather than NHEJ. This finding was further confirmed by the significantly reduced/increased RAD51 foci in NDRG1 low-expressing/overexpressing SiHa cells (Fig. 5E, F). To further verify the effect of NDRG1 on the HR repair, we detected the expression levels of DNA repair proteins binding to chromatin after knocking down or overexpressing NDRG1(Fig. 5C, D). We found that silencing NDRG1 inhibits the protein levels and phosphorylation of key HR repair proteins (RAD51, ATR, BRCA1, and CHK1), whereas overexpression of NDRG1 increases both expression and phosphorylation levels of the HR-related proteins. We further validated the expression of HR repair proteins after overexpressing (or silencing) NDRG1 in NSUN6-knockdown (or NSUN6-overexpression) SiHa cells by WB analysis (Fig. 5G-H). We found the expressions and phosphorylation of the key HR proteins significantly decreased after NSUN6 knockdown in SiHa cells, which could be rescued by stable overexpression of NDRG1 (Fig. 5G). Consistent with the above results, upregulated NSUN6 resulted in elevated expression and phosphorylation levels of the HR proteins (Fig. 5H), and silencing of NDRG1 neutralized the effects of NSUN6 overexpression (Fig. 5H). These results strongly support that NSUN6 and NDRG1 participate in DDR through the HR pathway, revealing a novel mechanism for cervical cancer radioresistance.

### NDRG1 overexpression dampens the radiosensitizing effect of NSUN6 knockdown in cervical cancer

**Fig. 6:**
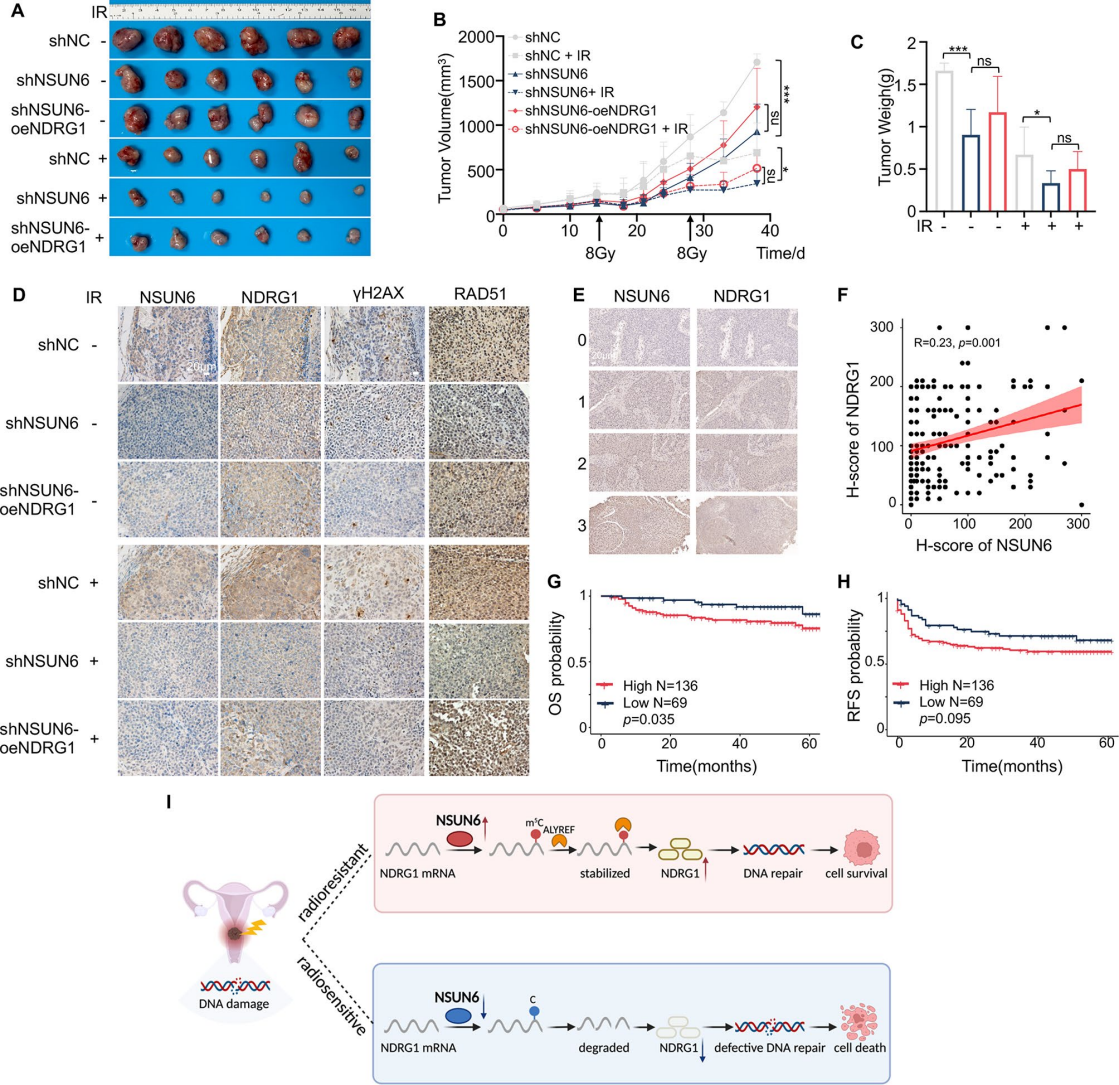
The roles of NSUN6-ALY-m^5^C-NDRG1 axis in cervical cancer radioresistance. (**A**-**C**) Representative images (**A**), growth curves (**B**), and weight curves (**C**) of xenograft tumors derived from SiHa-shNC cells, SiHa-shNSUN6 cells, and SiHa-shNSUN6-oeNDRG1 cells in nude mice. (**D**) Sections of tumors were stained with anti-NSUN6, anti-NDRG1, anti-γH2AX, and anti-RAD51 antibodies by IHC. (**E**) expression of NSUN6 and NDRG1 by IHC staining of the cervical cancer tissue arrays. (**F**) IHC staining showed that NSUN6 expression was positively correlated with NDRG1 expression. (**G**-**H**) Kaplan-Meier analysis of the overall survival and recurrence-free survival of 205 cervical cancer patients stratified according to NSUN6 expression level. All of those patients with advanced cervical cancer received standard radical radiotherapy. (**I**) The proposed model for the regulatory landscape of the NSUN6/ALYREF-m^5^C-NDRG1 signaling axis in promoting radioresistance in cervical cancer. Created in BioRender.com. A two-sided Student’s t-test was used for statistical analysis (**B**-**C**). *p < .05, ***p < .001. A log-rank test is used for statistical analysis in F. OS, overall survival; RFS, recurrence-free survival

To investigate if overexpressing NDRG1 decreases radiosensitivity induced by NSUN6 knockdown, we transfected NDRG1-overexpressing plasmids into NSUN6-knockdown SiHa and Me-180 cells for in vitro functional validation (Fig. S4A). As expected, overexpressing NDRG1 rescued the proliferation ability of NSUN6 knockdown cells (Fig. S4B). Elevated NDRG1 weakened the inhibitory effect of NSUN6 knockdown on the clonogenic ability after IR (Fig. 5I). Furthermore, flow cytometry results revealed that upregulation of NDRG1 significantly promoted IR-induced cell apoptosis in NSUN6-downregulated cells (Fig. 5G). Moreover, activation of NDRG1 markedly inhibited radiotherapy-induced DNA damage in NSUN6 knockdown cells (Fig. 5K). We also established SiHa-cell-derived xenograft models to validate the functions of NSUN6 and NDRG1 in vivo. The results indicated that the suppression of NSUN6 significantly hindered tumor growth with or without radiation. However, the restoration of NDRG1 counteracted the inhibitory effect of NSUN6 downregulation on tumor growth (Fig. 6A-C). In addition, IHC results showed that RDA51 (an HR repair biomarker) expression decreased in tumor tissues of the NSUN6 knockdown group compared with the control group but increased in the NDRG1 rescued group (Fig. 6D), which agreed with the results illustrated in Fig. 5E. Moreover, we observed an increase in NSUN6 expression in SiHa-CDXs following irradiation compared to those that did not receive irradiation (Fig. 6D). These above findings demonstrated that NSUN6 promotes cervical cancer radioresistance by regulating the expression of NDRG1.

### NSUN6 overexpression predicts poor OS and PFS in cervical cancer

Finally, we characterized the relationships between NSUN6/NDRG1 expression and clinical prognosis in cervical cancer by performing IHC in 205 advanced cervical cancer tissues. We found a positive correlation between the expression of NSUN6 and NDRG1 in cervical cancer (Fig. 6E-F) consistent with the results illustrated in Fig. 3D-I. According to the optimal cutoff point calculated by the Kaplan-Meier Plotter online database, samples with an H-score of no more than 20 were labeled as the low NSUN6 expression population, and the rest as the high NSUN6 expression population. KM-plotter analysis demonstrated that lower NSUN6 expression predicted longer OS (*p* = .035, Fig. 6G) and longer RFS (*p* = .096, Fig. 6H). However, NDRG1 was not an independent predictor of OS and RFS in cervical cancer (Fig. S4C). By joint analysis of the expression of NSUN6 and NDRG1, we found that cases with high expression of both NSUN6 and NDRG1 tended to exhibit a worse prognosis in cervical cancer (Fig. S4D).

## Discussion

Cisplatin-based chemo-radiotherapy is the standard first-line treatment for advanced cervical cancer patients, but approximately 30% of patients demonstrate intrinsic resistance to radiation [3]. Increasing evidence suggests that abnormal RNA modification can induce radioresistance of cancer cells. For example, YTHDF2 (m^6^A reader) overexpression drives extrinsic radioresistance of tumor cells [34]. The methyltransferase-like 14 (METTL14) promotes cancer stem cell persistence and mediates radioresistance in esophageal cancer [35]. In addition, chemical inhibitors targeting the oncogenic m^6^A regulators have been proven to effectively inhibit tumor cell proliferation and enhance the radiosensitivity of cancer cells [36]. To date, the first inhibitor targeting RNA modification, STC-15, a selective METTL14 inhibitor, has been approved to enter phase I clinical trials (NCT05584111). However, the roles of dynamic RNA modification in cervical cancer radiosensitivity remain unclear. Interestingly, the study by Chen Y et al. [21]. showed that upregulation of m^5^C-modified LRRC8A promoted cervical cancer tumorigenesis and elucidated the NSUN2-m^5^C-LRRC8A axis as a potential therapeutic target in cervical cancer, providing strong evidence to support our study. In the study, we demonstrated that the m^5^C level is significantly increased due to the upregulation of methyltransferase NSUN6 in radioresistant cervical cancer samples. Mechanistically, the overexpression of NSUN6 stimulates the m^5^C modification of NDRG1 mRNA, and the m^5^C reader ALYREF then directly binds to the m^5^C site on NDRG1 mRNA and maintains the stability of the mRNA. Elevated NDRG1 enhances DNA damage repair, which promotes the radioresistance of cervical cancer and leads to a worse clinical prognosis (Fig. 6I).

By LC-MS/MS analysis, we identified higher RNA methylation modifications such as m^5^C, m^6^A, and m^7^G in radioresistant cervical cancer samples. Consistent with our study, Xu et al. showed that high m^6^A abundance was associated with treatment resistance [24]. Moreover, overexpression of NSUN6, an m^5^C methyltransferase, was observed in radioresistant cervical cancer samples. Patients with high NSUN6 expression in cervical cancer had significantly worse OS and RFS. Next, we further verified this conclusion in a 3D bioprinted CC PDO model. By detecting NSUN6 expression levels in cervical cancer samples by IHC, we can prospectively predict the radiosensitivity of the corresponding patients. In accordance with clinical data, the knockdown of NSUN6 suppressed radiosensitivity in vivo and in vitro. Knockdown of NSUN6 promoted apoptosis and cycle arrest in SiHa and Me-180 cells and inhibited proliferation and DDR. Meanwhile, the downregulation of NSUN6 markedly suppressed tumor growth in the CDX model with or without radiation. Previous studies have shown that abnormal m^5^C modification can trigger treatment resistance of cancer cells by enhancing HR pathways [37], promoting the transcription of oncogenic genes [38], increasing glucose metabolism [39], lipid peroxidation [40], and ferroptosis [41]. Furthermore, NSUN6 was reported to exert oncogenic functions in urothelial bladder carcinoma [42] and lung cancer [43]while playing a tumor-suppressing role in pancreatic cancer [44]. Our data and published results suggested that m^5^C and NSUN6 may drive radioresistance in cervical cancer, but the exact mechanism requires further exploration.

Remarkably, we uncovered NDRG1 as a key downstream target of NSUN6 in cervical cancer through RNA-seq and MeRIP-seq. NDRG1 has been reported to be involved in numerous cancer processes, including epithelial-mesenchymal transition (EMT), cell migration, angiogenesis, and drug resistance [30]. Apart from its potent anti-metastatic role, NDRG1 has also been reported to play a pro-oncogenic role in many malignancies [45–47], including hepatobiliary, breast, and cervical cancers, and was associated with poor prognosis. The oncogenic effects of NDRG1 have been reported to relate to the mTOR signaling pathway and lipid metabolism [32, 45]. Consistently, our data indicated that silencing NDRG1 has a tumor-suppressive effect in cervical cancer by inhibiting cell proliferation and promoting apoptosis.

The main mechanism of radiotherapy is inducing double-stranded DNA break (DSB), and the increased DNA repair is the main cause of the radioresistance of cancer cells. Recent studies suggested that NDRG1 positively regulates and stabilizes DNA repair enzymes MGMT, APEX1, and PKNP, thereby driving temozolomide (TMZ) resistance in human glioblastoma and acute lymphoblastic leukemia (AML) [32, 33]. In this study, we first revealed that the downregulation of NDRG1 increases DNA damage and radiosensitivity in cervical cancer cells. Our data, including an HR/NHEJ reporter assay, RAD51 foci, and IHC staining in CDX tissues, demonstrated that NDRG1 enhances HR-mediated DDR in cancer cells. Previous studies have reported that NDRG1 stabilizes downstream proteins via direct protein-protein interactions [32]. Thus, we speculated that NDRG1 may promote DNA repair in cervical cancer by recruiting and stabilizing HR-related proteins, though the mechanism still requires further verification. HR repair is a precise DSB repair pathway that maintains genome integrity. The current study found that inhibiting HR repair can effectively inhibit tumor cell proliferation and increase sensitivity to IR [28]. The CHARIOT study demonstrated that the ATR inhibitor (Berzosertib) combined with chemoradiotherapy is feasible and well tolerated in oesophageal cancer patients [48]. More clinical trials of HR repair inhibitors combined with radiotherapy are in progress, including ATR, ATM, and RAD51 inhibitors. The critical role of NDRG1 in HR repair provides a new research direction for the radiosensitization of cervical cancer.

Separately, we found that the elevated NSUN6 can methylate NDRG1 mRNA at the 3’UTR region (with CDCC motif), thereby enhancing NDRG1 mRNA expression. However, the regulatory effects of RNA modification usually require interpretation from its reader. ALYREF and YBX1 are known m^5^C readers that recognize m^5^C-labeled mRNAs and promote their export, transcription, and stabilization [16, 17]. By RIP assay, we identified ALYREF specifically recognizing m^5^C-modified NDRG1 mRNAs. Moreover, ActD assays confirmed that knockdown of NSUN6 or ALYREF shortened the half-life of NDRG1 mRNA while overexpression of NSUN6 or ALYREF prolonged the half-life of NDRG1 mRNA. Collectively, our data provided novel evidence that NSUN6/ALYREF-mediated m^5^C modification maintained the stability of NDRG1 mRNA in cervical cancer. Further study of the detailed mechanism of the NDRG1 mRNA stability and translation regulated by the NSUN6-ALYREF signaling is warranted.

## Conclusions

In summary, we provided compelling in vitro and in vivo evidence demonstrating that m^5^C and NSUN6 can regulate the radiosensitivity of cervical cancer via the regulation of NDRG1. The methylation of NDRG1 mRNA by NSUN6 can increase the stability of NDRG1 mRNA via a special reader, ALYREF. Mechanistically, the NSUN6-m^5^C-NDRG1 axis promotes radioresistance of cervical cancer via increased DNA repair. Moreover, NSUN6 expression significantly increases in radioresistant samples and is correlated with poor prognosis in patients with cervical cancer. Therefore, NSUN6 may represent a potential predictor and therapeutic target for cervical cancer.

### Electronic supplementary material

Below is the link to the electronic supplementary material.

Supplementary Material 1

Supplementary Material 2

Supplementary Material 3

Supplementary Material 4

Supplementary Material 5

Supplementary Material 6

Supplementary Material 7

Supplementary Material 8

Supplementary Material 9

Supplementary Material 10

## Data Availability

No datasets were generated or analysed during the current study.

## Abbreviations

CC: Cervical cancer
m^5^C: 5-methylcytosine
m^6^A: N^6^-methyladenosine
m^7^G: 7-methylguanosine
m^3^C: 3-methylcytidine
m^1^A: N^1^-methyladenosine
ac^4^C: N^4^-acetylcysteine
m^5^U: 5-methyluridine
hm^5^C: 5-hydroxymethylcytosine
RMP: RNA-modifying proteins
LC-MS/MS: Liquid chromatography-tandem mass spectrometry
PDO: Patients-dervied organoid
MeRIP: Methylation RNA immunoprecipitation
NSUN6: NOP2/Sun RNA methyltransferase family members 6
ALYREF: Aly/REF export factor
YBX1: Y-box binding protein 1
NDRG1: N-myc downstream-regulated gene 1
RT: Radiotherapy
IR: Irradiation
IHC: Immunohistochemistry
IF: Immunofluorescence
DSB: Double-stranded DNA break
DDR: DNA damage repair
HR: Homologous recombination
NHEJ: Non-homologous end joining
CDX: Cell-dervied xenografts
IGV: Integrative Genomics Viewer
OS: Overall survival
RFS: Recurrence-free survival
