# Supplementary figures and images for: NSUN6-mediated 5-methylcytosine modification of NDRG1 mRNA promotes radioresistance in cervical cancer
Source: Mol Cancer. 2024 Jul 5;23:139. doi: 10.1186/s12943-024-02055-2 (PMC11225205; doi:10.1186/s12943-024-02055-2)

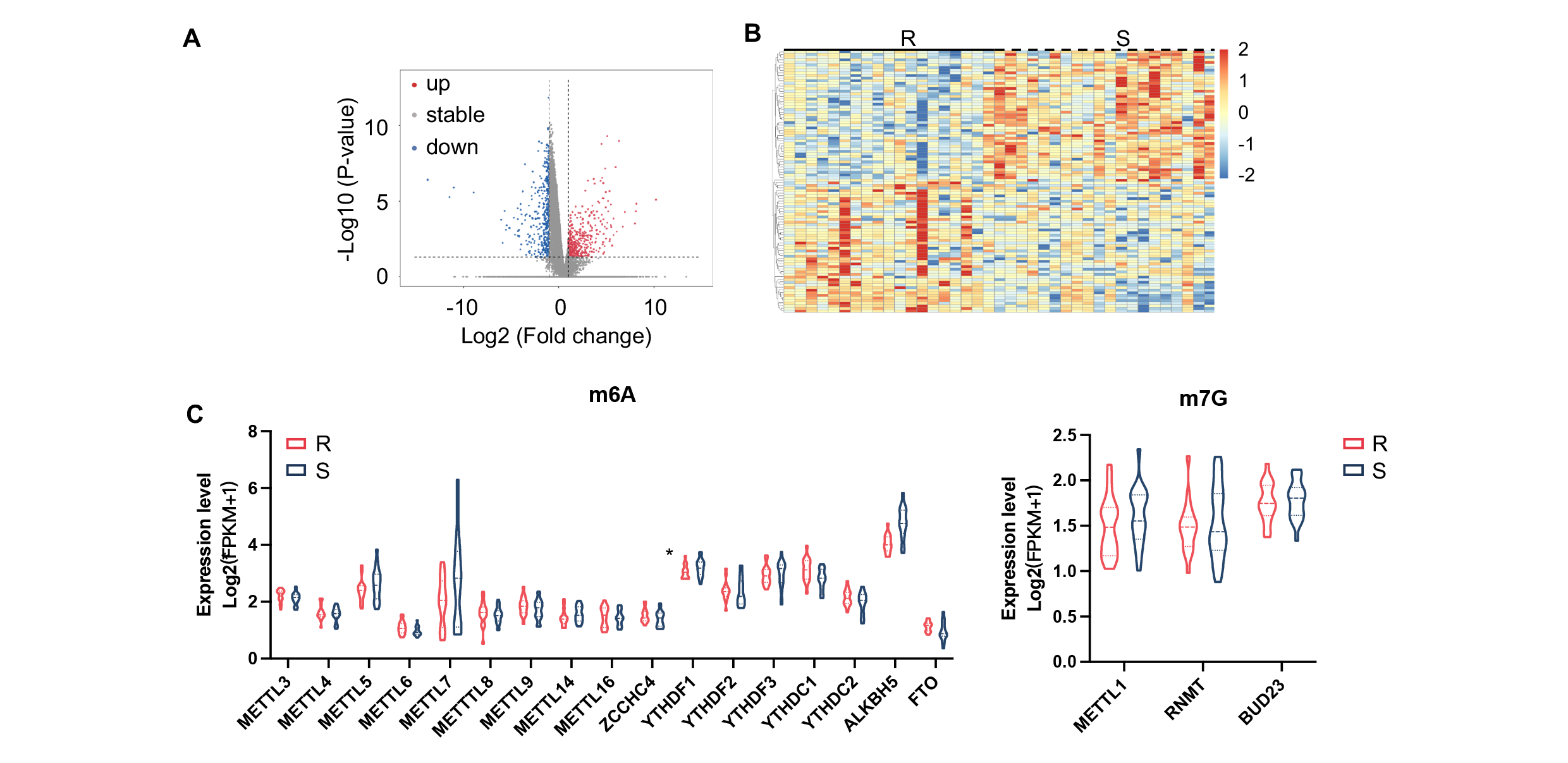

Supplement: Supplementary file 1 — Supplementary Material 1 [file 12943_2024_2055_MOESM1_ESM.tif]

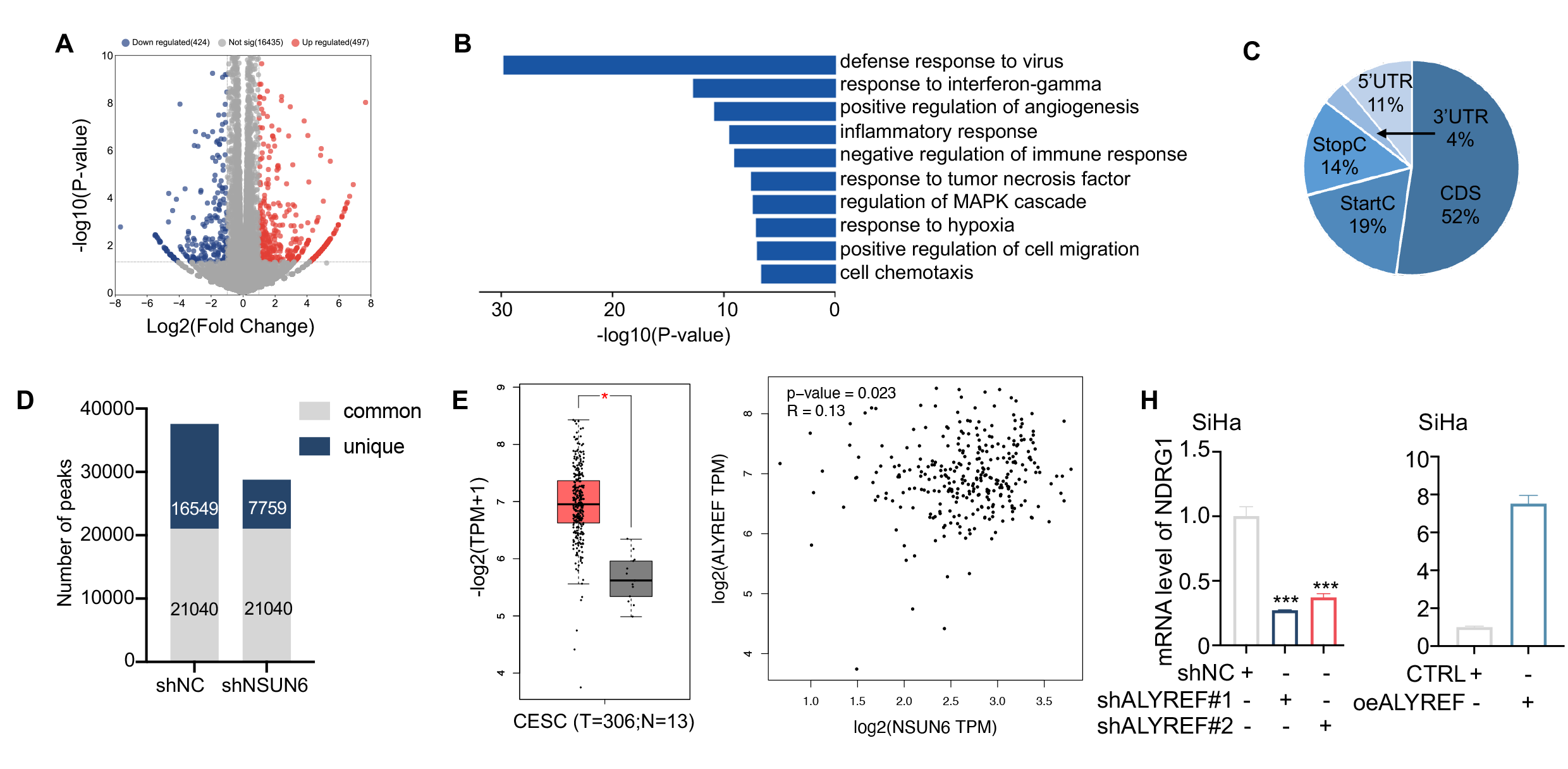

Supplement: Supplementary file 2 — Supplementary Material 2 [file 12943_2024_2055_MOESM2_ESM.tif]

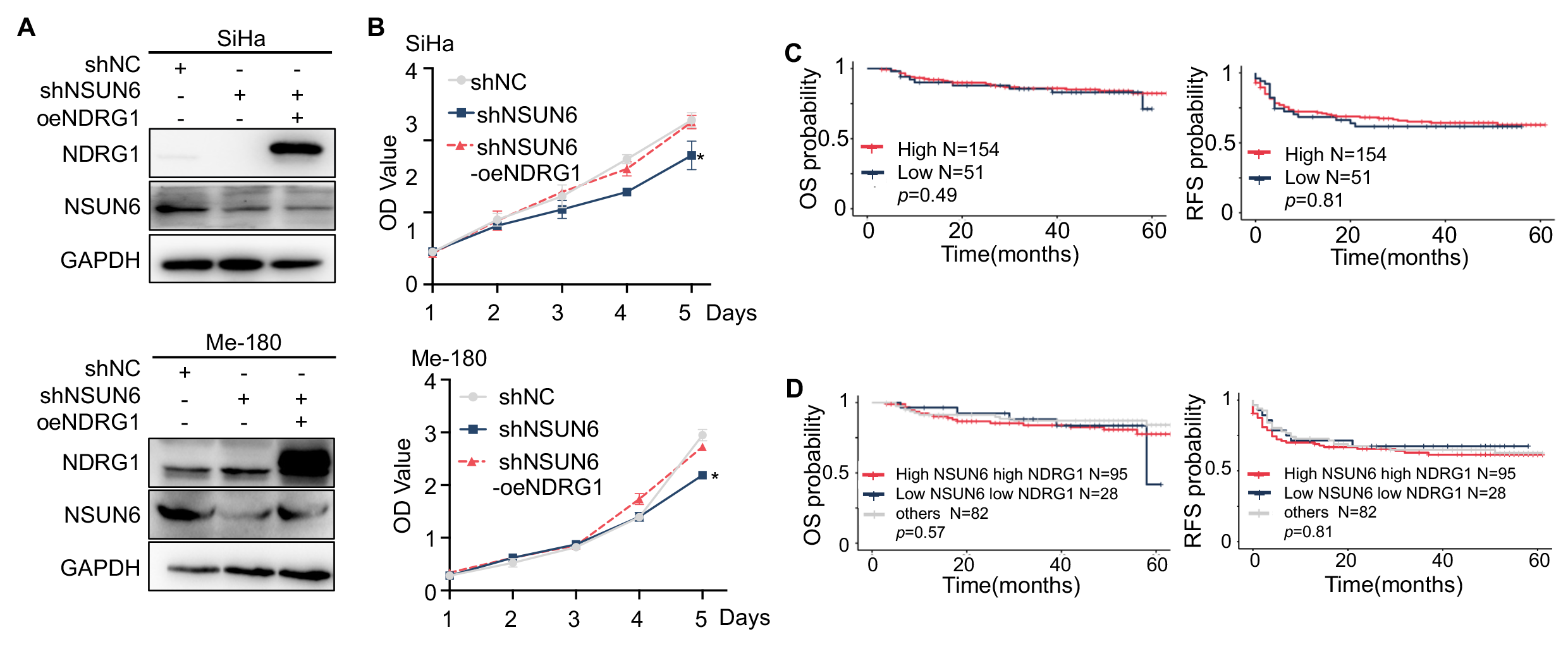

Supplement: Supplementary file 3 — Supplementary Material 3 [file 12943_2024_2055_MOESM3_ESM.tif]

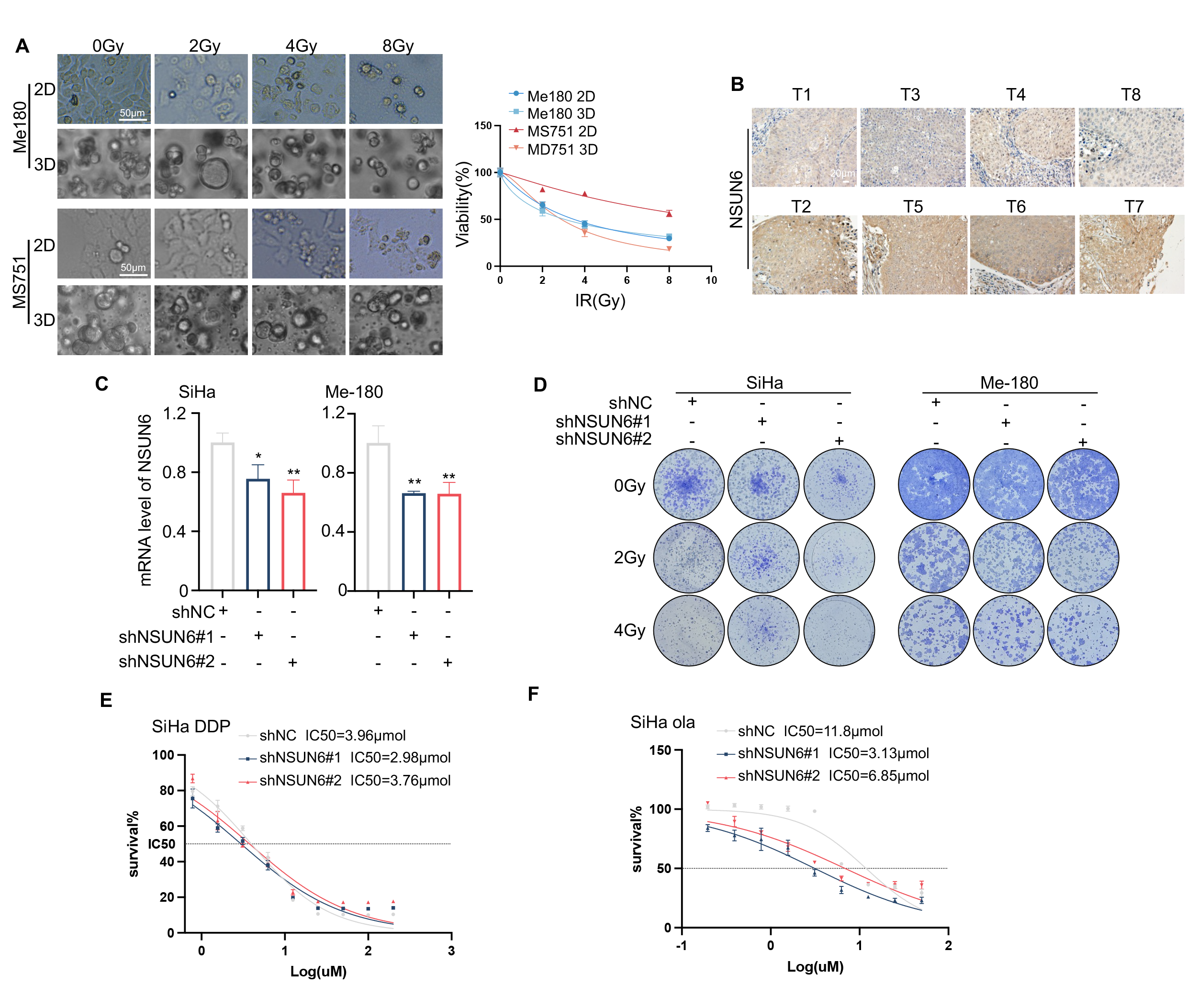

Supplement: Supplementary file 9 — Supplementary Material 9 [file 12943_2024_2055_MOESM9_ESM.tif]
