# Supplementary material for: NSUN6-mediated 5-methylcytosine modification of NDRG1 mRNA promotes radioresistance in cervical cancer
Source: Mol Cancer. 2024 Jul 5;23:139. doi: 10.1186/s12943-024-02055-2 (PMC11225205; doi:10.1186/s12943-024-02055-2)
Supplement: Supplementary file 4 — Supplementary Material 4 [file 12943_2024_2055_MOESM4_ESM.docx]

Table 1. Clinicopathological Features of the Overall Series

| **Characteristics** | **Resistant** | **Sensitive** |
| --- | --- | --- |
|  | No. (%) | No. (%) |
| All cases | 21 | 21 |
| Age (years) |  |  |
| Median (range) | 54 (43-71) | 55 (43-69) |
| FIGO stage |  |  |
| II | 5 (23.8) | 5 (23.8) |
| III | 10 (47.6) | 11 (52.4) |
| IV | 6 (28.6) | 5 (23.8) |
| Lymph node status |  |  |
| Positive | 10 (47.6) | 12 (57.1) |
| Negative | 11 (52.4) | 9 (42.9) |
| Parametrial invasion |  |  |
| Yes | 18 (85.7) | 20 (95.2) |
| No | 3 (14.3) | 1 (4.8) |
| Histology |  |  |
| SCC | 21 (100) | 21 (100) |

SCC, squamous cell carcinoma.
