# Supplementary material for: NSUN6-mediated 5-methylcytosine modification of NDRG1 mRNA promotes radioresistance in cervical cancer
Source: Mol Cancer. 2024 Jul 5;23:139. doi: 10.1186/s12943-024-02055-2 (PMC11225205; doi:10.1186/s12943-024-02055-2)
Supplement: Supplementary file 6 — Supplementary Material 6 [file 12943_2024_2055_MOESM6_ESM.docx]

Supplementary Table 3: siRNA sequence

| NSUN6 siRNA1-F | GACCCAGAAAGAAUAUUAATT |
| --- | --- |
| NSUN6 siRNA1-R | UUAAUAUUCUUUCUGGGUCTT |
| NSUN6 siRNA2-F | GUGUCAGCAUCACAAUUUATT |
| NSUN6 siRNA2-R | UAAAUUGUGAUGCUGACACTT |
| NDRG1 siRNA1-F | GGCCUGUCAUCCUCACCUATT |
| NDRG1 siRNA1-R | UAGGUGAGGAUGACAGGCCTT |
| NDRG1 siRNA2-F | CCUAACUCGAUUUGCUCUATT |
| NDRG1 siRNA2-R | UAGAGCAAAUCGAGUUAGGTT |
