# Supplementary material for: NSUN6-mediated 5-methylcytosine modification of NDRG1 mRNA promotes radioresistance in cervical cancer
Source: Mol Cancer. 2024 Jul 5;23:139. doi: 10.1186/s12943-024-02055-2 (PMC11225205; doi:10.1186/s12943-024-02055-2)
Supplement: Supplementary file 7 — Supplementary Material 7 [file 12943_2024_2055_MOESM7_ESM.docx]

RT-qPCR primers

| NSUN6-1 | F | TTAAGAGGAGCCCATGTCTATGC |
| --- | --- | --- |
|  | R | CTTTGCGGCTTAGTTCAGAAATC |
| NSUN6-2 | F | TCTCAGCCCTTCATTTGACAGT |
|  | R | TCCAGTGCTATAACTTCTCCCTG |
| NSUN6-3 | F | CAGAATGCCTTATTGTTAGGGCT |
|  | R | ACCATATCAAGTTTAACCGCCTT |
| Actin | F | CATGTACGTTGCTATCCAGGC |
|  | R | CTCCTTAATGTCACGCACGAT |
| GAPDH | F | TGCACCACCAACTGCTTAGC |
|  | R | GGCATGGACTGTGGTCATGAG |
| NDRG1-1 | F | CTCCTGCAAGAGTTTGATGTCC |
|  | R | TCATGCCGATGTCATGGTAGG |
| NDRG1-2 | F | GTCCTTATCAACGTGAACCCTT |
|  | R | GCATTGGTCGCTCAATCTCCA |
| NDRG1-3 | F | CCAACAAAGACCACTCTCCTC |
|  | R | CCATGCCCTGCACGAAGTA |
| ALYREF-1 | F | GCAGGCCAAAACAACTTCCC |
|  | R | AGTTCCTGAATATCGGCGTCT |
| ALYREF-2 | F | TATGATCGCTCTGGTCGCAG |
|  | R | AGAGGGACGCCGTTGTACT |
| ALYREF-3 | F | ACATTCAGCTTGTCACGTCAC |
|  | R | TCTAGTCATGCCACCTCTGTTTA |

MeRIP-qPCR

| NDRG1-1 | F | CGCATCCTCTTCCATTAACC |
| --- | --- | --- |
|  | R | TTGGCAGTTTGGTGTCTCTG |
| NDRG1-2 | F | GAGGGCACCCACGTAATAGA |
|  | R | ATGGAGGTCTCCTGCTAGGC |
| NDRG1-3 | F | ATTATTGGCATGGGAACAGG |
|  | R | TCTTCCTTCCCAAAAAGGTG |
