# Supplementary material for: NSUN6-mediated 5-methylcytosine modification of NDRG1 mRNA promotes radioresistance in cervical cancer
Source: Mol Cancer. 2024 Jul 5;23:139. doi: 10.1186/s12943-024-02055-2 (PMC11225205; doi:10.1186/s12943-024-02055-2)
Supplement: Supplementary file 10 — Supplementary Material 10 [file 12943_2024_2055_MOESM10_ESM.docx]

Supplementary Figure legends

**Supplementary Fig. 1.** (A) The volcano plot of differentially expressed genes in radioresistant and radiosensitive cervical cancer. Red indicates upregulation, blue downregulation, and gray non-dysregulation. (B) The heatmap of differentially expressed genes in radioresistant and radiosensitive cervical cancer. Intensity of color represents Log2(FPKM+1). (C-D) The expression of 17 m^6^Aregulators (C) and three m^7^G regulators (D) between radioresistant and radiosensitive CC samples were detected by mRNA sequencing. A two-sided Student’s t-test was used for statistical analysis (C). **p* < .05. m^6^A, *N*^6^-methyladenosine; m^7^G, 7-methylguanosine. R, radioresistant cervical cancer samples; S, radiosensitive cervical cancer samples.

**Supplementary Fig. 2.** (A) Dose-response to radiation of Me-180 and MS751 in 2D and 3D models. Representative bright-ﬁeld images on day four after irradiation(left), dose-response curves on day four after irradiation(right). (B) IHC staining images of NSUN6 in these eight samples. (C) qRT-PCR detected NSUN6 levels in SiHa and Me-180 knockdown cells. (D) Representative image of colony formation assay in SiHa and ME-180 treated with radiation of different doses. (E) Cisplatin (DDP) IC50 curves in NSUN6 knockdown cells and corresponding negative control (NC) cells. (F) Olaparib (ola) IC50 curves in NSUN6 knockdown cells and corresponding negative control (NC) cells.

**Supplementary Fig. 3**. (A) The volcano plot of differentially expressed genes in NSUN6 knockdown SiHa cells and corresponding control cells. Red indicates upregulation, blue downregulation, and gray non-dysregulation. (B) Enrichment of gene function of downregulated genes in NSUN6 knockdown SiHa cells compared to the control group (Metascape.org). (C) The normalized distribution of m^5^C peaks. (D) m^5^C-MeRIP sequencing showed the common and unique m^5^C peaks in NSUN6 knockdown SiHa cells compared to the control cells. (E) The level of NSUN6 expression was analyzed in CESC (N=306) and normal cervix tissues (N=13) by GEPIA2 (left). The relationship between NSUN6 and ALYREF in cervical cancer by GEPIA2. (H) NDRG1 mRNA levels in SiHa cells after ALYREF knockdown or overexpression. ****p* < .001; error bars represent means ± SD. A log-rank test is used for statistical analysis in F. CDS, coding sequence; UTR, untranslated regions; m^5^C, 5-methylcytosine.

**Supplementary Fig. 4.** (A) Western blotting examined the overexpression efficiency of NDRG1 in NSUN6 knockdown SiHa and Me-180 cells. (B) Effects of NDRG1 overexpressing on abilities of NSUN6 knockdown cell proliferation. (C-D) Kaplan-Meier analysis of OS and RFS in 205 cervical cancer cases stratiﬁed according to NDRG1 expression level(C) or NSUN6/NDRG1 expression (D). A log-rank test is used for statistical analysis in C and D. OS, overall survival; RFS, recurrence-free survival.
